# The Prognostic Value of Using Ultrasonography in Cardiac Resuscitation of Patients with Cardiac Arrest

**Published:** 2016-09

**Authors:** Ehsan Bolvardi, Seyyed Mohsen Pouryaghobi, Roohye Farzane, Niaz Mohamad Jafari Chokan, Koorosh Ahmadi, Hamidreza Reihani

**Affiliations:** 1 Department of Emergency Medicine, Mashhad University of Medical Sciences, Mashhad, Iran;; 2 Department of Anesthesiology, Alborz University of Medical Sciences, Karaj, Iran;; 3 Department of Emergency Medicine, Alborz University of Medical Sciences, Karaj, Iran

**Keywords:** Cardiac Ultrasonography, Cardiac arrest, Cardiopulmonary Resuscitation

## Abstract

Cardiopulmonary arrest is the final result of many diseases and therefore, need for a careful implementation of cardiopulmonary resuscitation (CPR) protocols in these cases is undeniably important. The introduction of ultrasound into the emergency department has potentially allowed the addition of an extra data point in the decision about when to cease cardiopulmonary resuscitation (CPR). The aim of this study is to evaluate the ability of cardiac ultrasonography performed by emergency physicians to predict resuscitation outcome in adult cardiac arrest patients. Ultrasonographic examination of the subxiphoid cardiac area was made immediately after admission to the emergency department with pulseless cardiac arrest. Sonographic cardiac activity was defined as any detectable motion within the heart including the atria, ventricles or valves. Successful resuscitation was defined as: return of spontaneous circulation for ≥ 20 min; return of breathing; palpable pulse; measurable blood pressure. The present study includes 159 patients. The presence of sonographic cardiac activity at the beginning of resuscitation was significantly associated with a successful outcome (41/49 [83.7%] versus 15/110 [13.6%] patients without cardiac activity at the beginning of resuscitation). Ultrasonographic detection of cardiac activity may be useful in determining prognosis during cardiac arrest. Further studies are needed to elucidate the predictive value of ultrasonography in cardiac arrest patients.

## INTRODUCTION

Cardiopulmonary arrest is the final result of many diseases and therefore, need for a careful implementation of cardiopulmonary resuscitation (CPR) protocols in these cases is undeniably important. Over the last fifty years early detection of cardiac arrest and CPR have saved lives of hundreds of thousands of people worldwide, which indicates the importance of research on this specific topic (1). It is recommended that any patient with cardiopulmonary arrest must first be assessed and treated in accordance with advanced cardiovascular life support (ACLS) and basic life support (BLS) guidelines, and be studied for treatable and reversible causes of cardiac arrest (2).

Lack of pulse during CPR does not always mean death, because ineffective contractions of the heart may still exist which means that there is still chance of treatment and resuscitation (3, 4). Therefore diagnostic and prognostic measures should always be take into consideration, because they increase control of the team on the revival of a particular patient. For example, a heart ultrasound is a vital diagnostic tool, which is increasingly use in emergency situations, and can be an effective diagnostic tool in cardiac arrest, especially for evaluation of the presence or absence of cardiac movements (5, 6).

Cardiac ultrasound could provide data in relation with heart contractions in patients without a pulse and is independent of the heart rhythm of the patient. Early identification of cardiac contractions in patients without a pulse may provide information for prognosis of resuscitation. Despite intensive studies on the evaluation of prognostic value of cardiac ultrasound for prediction of successful outcome of CPR, there is no general agreement on this issue (7).

Cardiac ultrasound has shown to be successful in the diagnosis and assessment of associated lesions and reversible causes of cardiac arrest (cardiac tamponade, mass lesions such as clots or tumor shrinkage, assessment of ventricular volume and left ventricular regional wall motion of the heart, etc) and performing therapeutic decisions (8).

The aim of this study is to evaluate the prognostic value of cardiac ultrasound and assessment of primary cardiac rhythm in stratification of patients with cardiac arrest.

## MATERIALS AND METHODS

In this study, 159 people that according to the American Heart Association (AHA) guideline (including lack of responsiveness of the patient, no breathing and/or occasionally interrupted breathing) had cardiac-respiratory arrest were undergone CPR based on ACLS protocol. All of the patients who were admitted to the emergency department of Imam Reza Training and Research Hospital, were evaluated and treated by emergency physicians. Initial rhythm of the patient (including asystole, ventricular tachycardia, ventricular fibrillation and pulseless electrical activity) was recorded by the physician using AED defibrillator instrument. At the same time, another physician who was not a member of the resuscitation team and had no knowledge of initial rhythm of patient conducted the cardiac ultrasonography using Curvilinear probe (Honda-japan) and Subxiphoid approach (due to its minimal interference with resuscitation). The resulting four chamber view was carefully evaluated by the physician and the presence of any heart activity including the ventricles, galleries, valves, etc were examined during cardiac ultrasound.

The positive outcomes were considered to be any successful retention of blood flow for more than 20 minutes, returning of breathing (excluding gasping, coughing or sudden movements), evidence of a palpable pulse and measurable blood pressure. At the end, the predictive value of cardiac kinetic activity in ultrasound evaluation as an early determiner for prognosis of cardiorespiratory arrest was examined.

In this study adult patients (more than 18 years) with traumatic cardiopulmonary arrest inside or outside the hospital with non-traumatic causes were included in the study and patients less than 18 years, patients with end-stage chronic diseases (including metastatic cancers and hematologic cancers) spend, drowning, with stroke, severe hypothermia (body temperature less than 30°C) were excluded from the study. Data and statistical analysis was performed by SPSS software and independent student t-test.

## RESULTS

The study included 159 patients of the health center at Imam Reza Hospital in Mashhad city. All of the patients, no call and no central pulse are in need of CPR. Demographic data of the patients, according to their arrival cardiac rhythm, are shown in Table 1. Out of the 159 patients, 76 (47.8%) were females and 83 (52.2%) were males. The average age of the patients was 57.11 ± 14.97. Regarding the possible cause of CPA, in 100 patients (62.9%) was non-traumatic and in 59 patients (27.1%) was traumatic.

**Table 1:** Clinical and demographic characteristics of the adult patients with cardiac arrest

Clinical and demographic characteristics		Total number (n=159)	Successful resuscitation (NO/%)	Unsuccessful resuscitation (NO/%)	*P* Value

Gender	Male	76	29 (38.2)	47 (61.8)	0.45
	Female	83	27 (32.5)	56 (67.5)	
Age	18-40	22	4 (18.2)	18 (81.8)	0.07
	>40	137	52 (28)	85 (62)	
Arrest location	In hospital	46	26 (56.5)	20 (43.5)	<0.001
	Out of hospital	113	30 (26.5)	83 (73.5)	
Initial arrest rhythm	Asystole	79	30 (60)	20 (40)	<0.001
	VF/pVT	30	12 (15.2)	67 (84.8)	
	PEA	50	14 (46.7)	16 (53.3)	
Cause of Arrest	Traumatic	59	9 (15.3)	50 (84.7)	<0.001
	Non-Traumatic	100	47 (47)	53 (53)	

When we examined the cardiac rhythm on arrival, 79 patients had asystole, 30 patients had ventricular fibrillation/pulseless ventricular tachycardia (VF/pVT) and 50 patients had pulseless electrical activity (PEA). There was no significant difference in terms of the average age of men and women (*P*=0.45) according to rhythms of arrival.

The success of resuscitation of patients with PEA, asystole and VF/pVT rhythm was 30 (60%), 12 (15.2%) and 14 (41.7%) respectively. Unsuccessful resuscitation of patients with PEA, asystole and VF/pVT rhythm was 20 (40%), 67 (84.8) and 16 (53.3) respectively.

Results of USG of the patients was as follows:

From total of 159 cases examined, in 49 patient initial positive cardiac kinetic activity was detected and in 110 patient no activity was seen. Among the cases that had positive kinetic activity, 41 cases (83.7%) show successful response to resuscitation and 8 cases (16.3%) did not respond to resuscitation efforts. On the other hand, from 110 cases in which no heart activity was detected on ultrasound, only 15 (13.6%) had a successful response to resuscitation and in other 95 cases (86.4 percent) the efforts for resuscitation was not successful. The difference between successful and unsuccessful response to resuscitation was statistically significant (*P*=0.001).

## DISCUSSION

Cardiopulmonary arrest (CPA) is the main cause of death in many parts of the world in spite of significant improvements in treatment (9). CPA patients have a high mortality rate (10). Cardiopulmonary resuscitation (CPR) is the most important intervention that connects the CPA, to life (9). Usage of the bedside ultrasound (USG) in the emergency department by emergency medicine specialists began at the late 80s with trauma patients, and expanded rapidly over the last ten-years (11-14). USG is also used to detect the presence of cardiac activity during CPR and for invasive procedures (intubation, central/ femoral vein catheterization, etc.) (4, 15-17).

Salen et al, have studied the ability of cardiac USG in predicting outcome of patients with heart failure in 2004 (4). In this study, in order to evaluate presence or absence of kinetic activity of the heart, patients with PEA and asystole were undergone B-mode transthoracic USG during the resuscitation procedure. Several end points have been considered as the potential prognostic signs of resuscitation including: heart rhythm, detectable heart activity with USG and duration of pre-hospital and ED resuscitation. From 70 patients that have been examined in this study, the rhythm in 36 cases was asystole and in 34 cases was PEA. Results of this study shows that B-mode USG could help physicians to make right decision during CPR in CPA patients with asystole or PEA.

Blaivas et al have studied the prognostic value of USG during the initial assessment of patients requiring CPR (10). 169 patients were evaluated in this study. Results of this study showed that all patients without cardiac movement in USG have died regardless of their initial heart rhythm.

The present study was conducted on 159 patients requiring cardiopulmonary resuscitation. The aim of this study was to evaluate the ability of heart ultrasonography as a predictor of prognosis in patients with cardiac arrest. Our results show that finding heart activity using USG at the beginning of the process of rehabilitation in patients with heart failure is significantly correlated with successful resuscitation. This finding is consistent with results of Tomruk et al in which the presence of sonographic cardiac activity at the beginning of resuscitation was significantly associated with a successful outcome (19/27 [70.4%] versus 55/122 [45.1%] patients without cardiac activity at the beginning of resuscitation) (18). Our findings are also consistent with results of salen *et al*.

The primary goal of cardiac ultrasonography in patients with heart failure is to recognize organized contractions of the heart and thus improve subsequent rehabilitation program in these patients. USG, on the other hand, can be used to detect some common causes of heart failure, including embolism, cardiac tamponade or severe Hypovolemia.

Although sufficient evidence does not exist to support or refute the routine use of ultrasound to predict the success of resuscitation, but it has been proven that focused USG of heart can be used during cardiopulmonary resuscitation (7).

Several studies have investigated the role of ultrasound in determining the cause of heart failure and has been shown that the use of ultrasound can accelerate detection of causes of cardiac arrest and reduce the time required for treatment (8).

In this study, absence of heart activity on USG at the beginning of cardiopulmonary resuscitation is correlated with failure in resuscitation procedure and therefore shows that the absence of ultrasound represents a poor outcome in resuscitation of patients with heart failure in the emergency department. This result is in accordance with the study of Blaivas *et al* in which, regardless of the initial rhythm of patients, all of them who does not show cardiac movement in the initial USG were died (10). Therefore the positive predictive value in study of Blaivas et al was 100%, but in our study, considering the fact that 13.6% of patients without cardiac movement in the initial USG show positive response to CPR, the positive predictive value was 83.7%.

In the present study, USG showed sensitivity of 73.2%, specificity of 92.2%, negative predictive value of 84.6% and positive predictive value of 83.7%, that in comparison with study of Tomruk et al with sensitivity of 25%, specificity of 90%, negative predictive value of 60%, and positive predictive value of 70% has higher specificity and predictive value. Another difference between our study and study of Tomruk et al is inclusion of initial rhythm of patient in our study which is not included in Tomruk’s work.

### Limitations

The most important limitation of the study was the relatively small sample size. In addition, patients did not undergo conventional cardiac echocardiography.

Other limitations of this study are including short time of survival of patients in cardiopulmonary resuscitation (20 minutes after resuscitation was considered to be successful resuscitation), also long-term outcomes after resuscitation has not been studied in this research.

Since, the goal of the present study was to investigate the role of ultrasound in prognosis of patients with and without cardiac activity we did not elaborate on causes of cardiac arrest and confined our study on outcome of treatment regardless of the cause.
